# 3D-Powder-Bed-Printed Pharmaceutical Drug Product Tablets for Use in Clinical Studies

**DOI:** 10.3390/pharmaceutics14112320

**Published:** 2022-10-28

**Authors:** Korinde A. van den Heuvel, Alberto Berardi, Lisa B. Buijvoets, Bastiaan H. J. Dickhoff

**Affiliations:** DFE Pharma, Klever Strasse 187, 47568 Goch, Germany

**Keywords:** 3D printing, additive manufacturing, tablets, powder bed printing, lactose, clinical trials, starch, model compound, API

## Abstract

Printing of phase 1 and 2a clinical trial formulations represents an interesting industrial application of powder bed printing. Formulations for clinical trials are challenging because they should enable flexible changes in the strength of the dosage form by varying the active pharmaceutical ingredient (API) percentage and tablet mass. The aim of this study was to investigate how powder bed 3D printing can be used for development of flexible platforms for clinical trials, suitable for both hydrophilic and hydrophobic APIs, using only conventional tableting excipients. A series of pre-formulation and formulation studies were performed to develop two platform formulations for clinical trials using acetaminophen and diclofenac sodium as model compounds and lactose and starch as excipients. The results showed that the type of starch used as the formulation binder must be optimized based on the type of API. Moreover, powder blend flow and liquid penetration ability proved to be critical material attributes (CMAs) that need to be controlled, particularly at high drug loading. Optimization of these CMAs was performed by selecting the appropriate particle size of the API or by addition of silica. A critical process parameter that had to be controlled for production of tablets of good quality was the quantity of the printing ink. After optimization of both the formulation and process parameters, two platform formulations, that is, one for each API, were successfully developed. Within each platform, drug loading from 5 up to 50% *w*/*w* and tablet mass from 50 to 500 mg were achieved. All 3D-printed tablets could be produced at tensile strength above 0.2 MPa, and most tablets could enable immediate release (i.e., >80% *w*/*w* within 30 min).

## 1. Introduction

3D printing is an emerging technology that can support introduction of “Industry 4.0” within the pharmaceutical industry [1]. Application of 3D printing could solve various challenges, such as preparing formulations containing multiple active pharmaceutical ingredients (API) [2,3,4]. Even though there are numerous proofs of principle in 3D printing in scientific literature [5,6,7], industrial application of 3D printing in pharma is still limited. Use of 3D printing applied on a large-scale to produce medicines seems to be hindered by the conservative approach of pharmaceutical companies towards medicine manufacturing. The transition from conventional production technologies to 3D printing is only possible if 3D printing results in clear benefits over traditional technologies [8,9,10,11].

3D printing technologies may not yet be ready to replace large-scale conventional manufacturing technologies (e.g., tableting). Nevertheless, as observed in the food industry [12,13], the strength of 3D printing lies in creating small batch sizes of products with varying compositions or other product aspects. This flexibility of 3D printing could be beneficial in development of pharmaceutical formulations for clinical trials, which typically require small batch sizes and numerous tablet doses. As such, 3D printing can overcome the economic challenge of producing batches for clinical trials using conventional tableting techniques.

Formulation development for a clinical study can be approached in various ways [14], each with its own benefits and challenges. One option is to adjust the tablet dose by varying the tablet mass with a constant API percentage. A constant proportion of ingredients in the formulation could be beneficial for reducing the number of required release tests [15]; however, in blinded studies, tablets of varying mass might not be desirable. Therefore, production of tablets of constant mass with varying API percentages may be preferred. Each approach (i.e., a constant percentage of API or constant mass) requires its own formulation development. The versatility of 3D printing can accommodate tablet production using both approaches.

3D-printed tablets can be fabricated using various techniques [16]. The most commonly used methods are fused deposition modelling (FDM), direct melt extrusion (DME), selective laser sintering (SLS), and powder bed printing (PBP). FDM is a perfect fit for producing single tablets, making it an interesting technique for personalized medicine [17]. Finding suitable printable pharma-grade polymers remains a challenge. SLS has the advantage of having a higher printing resolution and creating tablets with lower friability, but it involves use of a laser, which could potentially degrade the API [18]. Powder bed tablet printing is, in essence, wet granulation on a powder particle scale and is a robust and established method of agglomerating particles. Using powder bed printing, a thin layer of powder is dispersed on the printing platform. The nozzle precisely jets small droplets of printing ink onto the powder in a predetermined shape. A new layer of powder is added to the printing bed and the wetting process is repeated. The tablet is finalized when a predetermined number of layers is added to the printing table. API can be added to the powder bed as well as to the printing ink. The powder bed printing technique is scalable as it has already been applied in the full-scale GMP production line of ZipDose technology from Aprecia [19].

Clinical trial formulations should contain safe and approved excipients. In addition, the formulation should readily release the API so that it can be solubilized and absorbed. Lactose and starch are among the safest and most widely used filler binders in the pharmaceutical industry, respectively. Sen et al. (2021) showed that powder properties are particularly important in powder bed printing [20] because tablets are created without a compression step. Flowability is a critical material attribute [21,22] for formation of powder beds without defects. Quick absorption of the ink is another important attribute as inhomogeneous absorption of ink can result in balling (rolling of the ink on the surface). In addition to the powder properties, print settings also play a major role in the quality of the final tablets [23,24]. For example, the type and amount of print ink influence the characteristics of the final tablet. Therefore, good tablet properties can only be obtained by balancing the delicate interplay between formulation and print settings in the process.

Formulation selection and establishment of optimized print settings in powder bed tablet printing have not received much attention in the scientific literature. More recent publications have addressed this topic, including Chang et al. (2021), who used a lactose, Kollidin^®^ VA 64 powder mixture in combination with a fixed API percentage [25]. Antic et al. (2021) assessed excipient powder placebo blends on printability [22], and Kozakiewicz-Latala et al. (2022) studied formulation of challenging medicines, such as hydrophobic drugs [26]. 3D-printed formulations used in clinical trials should be flexible and, therefore, allow for easy adjustment of tablet mass and/or drug loading. In addition, the tablet should have sufficient strength for adequate packaging, and the formulation should release the API quickly and completely. However, to date, only limited attention has been paid to use of powder bed tablet printing and its challenges in early phase formulation development in phase 1 and 2a clinical trials.

The aim of this study was to investigate whether lactose-based platform formulations could be produced into tablets with varying API doses via powder bed tablet printing for application in clinical trials. A lactose-based formulation was screened for its suitability for both hydrophilic and hydrophobic APIs. Initially, formulation optimization was performed by selecting the binder grade (partially pregelatinized starch) that would yield the best compromise of high tablet hardness while maintaining rapid dissolution. Then, the particle size distribution (PSD) of the model API and use of silica were investigated to ensure that the blends would have the required flow and print ink penetration times. Both parameters are prerequisites for efficient printability. Consequently, the process parameters for 3D printing were optimized by varying the amount of printing ink. Based on these screening results for formulation and process parameters, tablets ranging from 5 up to 50% *w*/*w* drug load and from 50 to 500 mg of mass were successfully created, and their dissolution behavior was investigated. This work demonstrates that immediate-release tablets with a broad range of drug loadings and weights can be prepared using powder bed printing. Thus, this technique can provide the flexibility of dosing and mass variation required for clinical trials.

## 2. Materials and Methods

### 2.1. Materials

Milled and sieved lactose monohydrate (DFE Pharma, Goch, Germany), partially pregelatinized maize starch grades 1 (less homogeneously gelatinized) and 2 (more homogenously gelatinized) (DFE Pharma, Goch, Germany), and colloidal silicon dioxide (Cabot Corporation, Boston, MA, USA) were used to create different formulations, as well as diclofenac sodium with a particle size of ×50 = 7 µm (Fagron, Capelle a/d IJssel, The Netherlands) and ×50 = 24 µm (Ofipharma, Ter Apel, The Netherlands), and acetaminophen fine powder with a particle size of ×50 = 21 µm (Tiefenbacher, Hamburg, Germany) and ×50 = 39 µm (Mallinckrodt, Raleigh, NC, USA).

### 2.2. Methods

#### Powder Mix Preparation

For all formulations, 20% *w*/*w* partially pre-gelatinized starch was used. The remaining 80% consisted of API (5 to 50%), lactose, and, when specified, 0.5% *w*/*w* silica. Addition of silica and/or variation in API concentrations was corrected with lactose.

The blends used for characterization were prepared by mixing the API and lactose for 15 min at 35 rpm using a Turbula T2F mixer, followed by sieving the API pre-blend (710 µm sieve). In the second step, the binder and disintegrant were added and mixed for 30 min at 35 rpm. In cases where silica was used, a pre-blend with silica and lactose at a 1:10 ratio was prepared, mixed at 35 rpm for 5 min, and sieved over a 710 µm sieve. The blends used for printing were created in a similar way, with the only difference that a 700 µm sieve was used for sieving and Stuart Scientific STR4 rotator drive unit with a drum and bottle holder was used to blend the mixtures.

### 2.3. Blend Characterization

A ring shear tester (Ring Shear Tester RST-XS, Dietmar Schulze, Wolfenbüttel, Germany) was used to determine the flow of the blends in duplicate. The flow is described by the flow function coefficient (FFC), which is the ratio of consolidation stress to yield strength. The blends were measured at 4 kPa pre-consolidation stress, and normal stresses of 1, 2.1, 3.2 kPa were used to shear to failure.

Bulk and tapped density were measured in duplicate according to USP <616>.

Particle size distribution (PSD) was measured in triplicate using a Helos/KR laser diffraction unit (Sympatec GmbH, Clausthal-Zellerfeld, Germany). Dry dispersion was measured at a pressure of 1.5 bar using an R5 Fourier lens with a 632.8 nm wavelength He-Ne laser as the light source.

Drop penetration time was measured using a drop shape analyzer (DSA) equipped with a powder sample holder (OCA 50 Dataphysics, Filderstadt, Germany). Ten microliters of print ink were dropped from 4 mm above the powder bed using an ESN16 dispense unit. Penetration time was recorded as the time between the moment the droplet hit the bed and the moment the droplet was fully adsorbed. Measurements were performed in sextuplicate.

### 2.4. Printing Process

**Figure 1:**
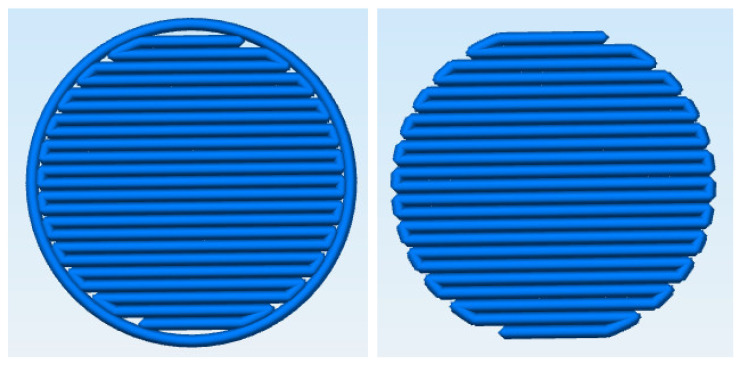
Print pattern used during the printing process. **Left**: an outer line is printed on the rim of tablet before the tablet is filled in line-wise direction. **Right**: no outer line is printed, and circle is being filled directly in a line-wise direction.

The formulations were printed using a powder bed printer (PBP) Next printer, developed at TNO (Eindhoven, The Netherlands) with a Lee valve INKA2476210H (0.178 mm). The powder blend was automatically dispensed from a hopper onto the powder platform and rolled out using a counter-rotating roller. A water/ethanol (95/5% *v*/*v*) ink solution was jetted onto the powder bed with varying line spacings (LS). Unless stated otherwise (Table 1), first, an outer line is printed, which is filled in a line-wise manner (Figure 1). Powder deposition and solution spraying were repeated until flat tablets with predetermined diameters and heights were created.

For screening purposes, tablets were printed using an adapted drop-shape analyzer (DSA). Powder blend addition and rolling were performed manually, and the water/ethanol (95/5% *v*/*v*) ink solution was jetted with the help of the DSA. These tablets were characterized with respect to their hardness and dissolution behavior. All measurements were performed in duplicate. This method was only applied if it was explicitly mentioned that the screening method was used, and all other tablets were printed using the TNO printer.

### 2.5. Tablet Characterization

Tablets were analyzed for weight, diameter, thickness, and hardness using an automated tablet tester (Sotax HT100, Lörrach, Germany). Tablet breaking force was measured at a constant speed of 2 mm/s, and the maximum force required to break the tablets was used as the crushing force. The measurements were performed in ten-fold.

Disintegration time was measured using an Erweka disintegration tester (Langen, Germany) with USP bolus-fluted plastic disks and demineralized water at 37 °C. Disintegration time was reported when the tablet disintegrated into particles small enough to pass through the mesh (pore size = 3 mm). Measurements were performed in six-fold.

Tablet dissolution was analyzed six-fold using a USP II dissolution tester (Vankel) in combination with a UV–Vis spectrophotometer (PerkinElmer Lambda 25)(Groningen, The Netherlands) at wavelengths of 243 nm (acetaminophen) and 276 nm (diclofenac sodium). Dissolution profiles were measured in 900 mL 0.05 M phosphate buffer pH 5.8 (acetaminophen) or 0.05 M phosphate buffer pH 6.8 (diclofenac sodium) at 37 °C with a paddle speed of 50 rpm.

## 3. Selection of Formulation and Print Settings

### 3.1. Formulation Optimization: Starch Selection

Pregelatinized starch acts as a tablet binder, which should ideally promote formation of hard tablets without negatively affecting disintegration [27]. Various grades of partially pregelatinized starches with different degrees and homogeneities of gelatinization are available in the market. These differences in gelatinization can affect hydration, binding, and disintegration properties of starch [28]. Van den Heuvel et al. (2021) found that a formulation based on partially pregelatinized starch combined with model compound diclofenac sodium could provide an optimal balance between high tablet strength and rapid API release [29]. In the current study, we investigated whether different grades of partially pregelatinized starch could improve the balance between hardness and dissolution.

Two experimental setups were used to study the characteristics of tablets containing different grades of starch. Initially, the hardness and drug release of tablets produced in-house using a modified drop shape analyzer (“Screening test” in Table 2) were measured. Then, the test was scaled up by repeating the measurements on tablets of the same composition but produced on a TNO 3D printer (“Tablets printed at TNO”).

Screening trials (Table 2) showed that starch type had an impact on hardness/dissolution balance of the printed tablets for both APIs. In the case of acetaminophen, starch grade 2 offered the best balance between binding and dissolution as it provided tablets twice as hard as starch grade 1 yet without a significant delay in dissolution. On the contrary, in the case of diclofenac sodium, starch grade 1 is preferred because it provided a similar binding effect (i.e., tablet hardness) but significantly faster dissolution than starch grade 2. This trend is noticed in both experimental settings (i.e., “Screening test” and “Tablets printed at TNO”). Starch grades 1 and 2 are different in that the former is less homogeneous and, hence, characterized by more clustered gelatinization than the latter. Presumably, owing to their different solubilities, the two APIs (acetaminophen is highly water-soluble; diclofenac sodium is poorly soluble) required a binder with different homogeneities of gelatinization to reach the required balance between tablet hardness and dissolution.

In conclusion, it was found that the different partially pregelatinized starches are not interchangeable. Therefore, starch grade 2 was selected as the binder of choice for acetaminophen and starch grade 1 for diclofenac sodium in subsequent formulation trials.

### 3.2. Formulation Optimization: Flow and Penetration Time

The important parameters for 3D powder bed tablet printing are powder flow and liquid penetration time. Easy-flowing powder with quick liquid penetration is required to obtain a powder bed without defects, in which the ink will be rapidly absorbed [30]. Blends that contain 30 to 50% *w*/*w* API with a small particle size distribution could potentially have poor flow and/or wettability. Therefore, to enable formulation of high-dosed 3D-printed tablets, API powder flow and wettability can be improved by modifying the particle size and/or adding a glidant. Therefore, this was investigated in the present study.

**Figure 2:**
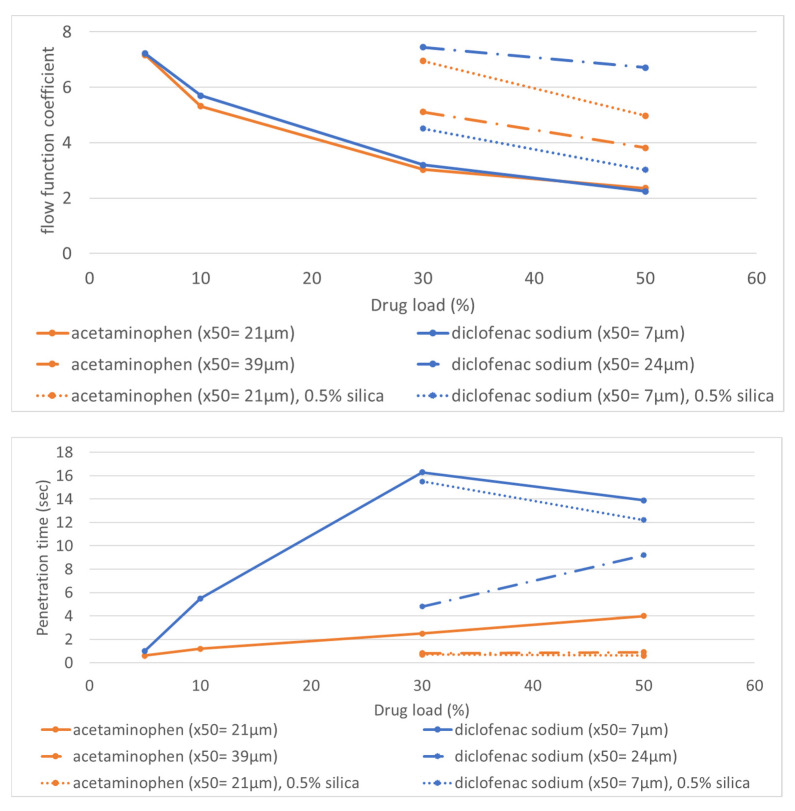
Flow expressed as FFC (**upper panel**) and penetration time measured via DSA (**lower panel**) at different drug load levels for both the hydrophilic and hydrophobic model compounds formulated with 20% *w*/*w* partially pregelatinized starch and lactose.

Figure 2 (upper panel) shows the flowability expressed as FFC for blends created with 5 up to 50% *w*/*w* drug load. Two different particle size grades of acetaminophen and diclofenac sodium were tested; for finer grades, addition of silica was also investigated. Increasing the particle size (from ×50 = 21 µm to ×50 = 39 µm for acetaminophen, and from ×50 = 7 µm to ×50 = 24 µm for diclofenac sodium) resulted, for both APIs, in improved flow, as expected. Addition of 0.5% *w*/*w* silica improved the flow of the acetaminophen blend but not that of the diclofenac sodium blend. The different effects of silica on the flow of the two APIs can be attributed to their different particle sizes (×50 = 21 µm for acetaminophen and ×50 = 7 µm for diclofenac sodium). It is possible that, in the case of cohesive micronized diclofenac, sodium agglomeration of the API occurred and the silica was only able to coat the agglomerates instead of the single particles. This inefficient coating could lead to limited flow improvement. As flow improvement by silica addition was dependent on the PSD of the API, in this study, silica was only added to formulations with a grade API with an ×50 above 20 µm.

The wettability of the same blends (5 up to 50% *w*/*w* drug load) was studied by liquid penetration time (Figure 2 lower panel). Acetaminophen formulations showed a penetration time of less than 5 s in all cases. Fine-grade diclofenac sodium formulations had penetration times longer than 5 s at drug loadings above 10% *w*/*w*, even after addition of hydrophilic silica. The use of a coarser grade of diclofenac sodium enabled quicker liquid penetration, possibly as a result of different packing, and, therefore, porosity of the powder. This is in line with previous research [29], where an increase in ×10 of the lactose/fully pregelatinized starch blend also resulted in quicker penetration. Based on the observed penetration times, it is expected that formulations with a higher drug loading of diclofenac sodium would be challenging to print owing to poor wettability. Therefore, it will be studied whether this issue can be solved by changing the print settings (Section 3.3).

The lactose/starch platform formulations were selected for printing at TNO based on the blend characterization described above. For acetaminophen, the same grade (×50 = 21 µm) was used for all drug loadings and silica was added to the formulation with 50% API. An alternative option to the use of silica, which was using a coarser API for 50% drug loading, was not explored. Addition of a glidant is a minor change in the platform, guaranteeing a high degree of consistency in acetaminophen performance throughout the different drug loadings. In addition, a change in the PSD of the API may have affected the functional properties of the tablets (e.g., dissolution). For diclofenac sodium, the fine (×50 = 7 µm) and coarse (×50 = 24 µm) grades of API were used for 5–10% and 30–50% drug loading, respectively. The coarser grade had to be chosen at high drug loading given that silica addition could not guarantee sufficient flow and wettability. The coarser grade was also suitable for printing lower drug loadings; however, this is not presented in this paper.

### 3.3. Print Settings: Varying the Amount of Print Ink

The starting point of this study was to print all platform formulations with one print setting to reduce the number of variables. As established in previous research [29], print settings should be developed in conjunction with formulation. There are various options for varying the amount of ink in the printing process. The most straightforward approach is to vary the line spacing, that is, by adjusting the distance between the droplets jetted on the powder bed. An alternative approach is to adjust the printing pattern. The starting print pattern of all formulations consisted of deposition of an outer circle, which was then filled line-wise (Figure 1). An alternative printing pattern is to remove the outer circle and print only the line-wise filling pattern. This approach would reduce the amount of printing liquid (Table 1) but could negatively impact the tablet strength as the edge of the tablet would be less consolidated. It must be noted that, during the study, tablets without an outer line were more difficult to measure in a classical tablet tester. These tablets had more irregular rims that would not break through a single fracture but were chipped into pieces during the tensile strength tests.

In order to select an appropriate starting point, the effect of varying the amount of print ink via line spacing was studied for the two formulations (10% *w*/*w* acetaminophen or diclofenac sodium) when printing a tablet with a diameter of 9 mm (Table 3). By decreasing the line spacing, the tablet mass of the acetaminophen formulation increased from 123 mg to 215 mg. Decreased line spacing leads to more bleeding during the printing process as more liquid penetrates below and beside the intended circle in the (first) layer, binding more powder to the tablet. Second, more liquid deposition results in more dissolved powder, and, hence, a gap forms on the printed surface. This gap is refilled when a new layer of powder is added to the print table; hence, more powder is added to the location of the printed circle, resulting in a mass increase. Varying the line spacing for the hydrophilic formulation from 0.43 down to 0.35 mm resulted in increased hardness and reduced dissolution. Further reduction in line spacing from 0.35 to 0.28 mm resulted in a decrease in release (93 to 78% *w*/*w* at 30 min) but no further increase in tensile strength (1.1 to 1.0 MPa). These outcomes were in line with the expectations; increasing the amount of liquid increased the consolidation until a plateau was reached. A line spacing of 0.35 mm provided an optimal balance between hardness and dissolution and was, therefore, selected as the print setting for all formulations.

For the diclofenac sodium formulation, printing was only possible at 0.35 mm line spacing (Table 3) because of the problematic wettability of the blend (see Figure 2). The lower line spacing overwetted the powder bed, leading to swelling of the outer line. Higher line spacing results in an extremely dry and rough surface.

Diclofenac sodium (×50 = 24 µm) blends at high drug loading (30 and 50% *w*/*w*) were difficult to print as the rim of the tablet overwetted and swelled, resulting in print defects, especially for tablets with a diameter of 12 mm. To avoid over-wetting, the amount of print ink was varied by adjusting the printing pattern. Printing with and without the outer line (Figure 1) resulted in tablets with comparable hardness and dissolution rates. There was a decrease in tablet mass from 134 to 117 mg for printing without an outer circle due to less wetted powder, and, hence, a smaller tablet diameter and mass. Removing the outer line of a tablet is an effective way to reduce the amount of printed ink without changing the line spacing. However, it must be noted that tablets with an outer line have a smoother appearance.

In conclusion, line spacing of 0.35 mm provided an optimal balance between hardness and dissolution at the 10% *w*/*w* drug load for the acetaminophen formulation. As the penetration time for drug loading up to 50% *w*/*w* is below 5 s (Figure 2), no challenges in wettability are expected, and a line spacing of 0.35 mm was, therefore, selected as the print setting for all formulations (Table 4). Diclofenac sodium formulations had longer penetration times (Figure 2) and could only be printed with a line spacing of 0.35 mm. The print pattern was adjusted in order to reduce the amount of print ink for the more challenging formulations (no outer line was printed for formulations with 30 and 50% *w*/*w*).

## 4. Printing Clinical Trial Formulations

The three most important parameters for clinical study development are tablet mass, strength, and API release during dissolution: tablet mass because it is directly related to API concentration, tablet strength because it is required to obtain the complete tablet packaged and dosed to the patient, and API release because it will indicate if the complete API is released and if the release is comparable for all formulations. Preferably, the different formulations had an API release of >80% *w*/*w* within 30 min and a tensile strength above 0.2 MPa (>0.2 MPa was taken as the target value as demonstration tablets from the powder bed printed technology produced on a full-scale GMP scale (ZipDose) had a strength of 0.15 MPa).

In this study, the tablet mass, tensile strength, and dissolution of the extremes (5 and 50% *w*/*w*) are presented. The complete dataset and photographs of all formulations provided in Table 4 can be found in the supplementary information.

### 4.1. Tablet Mass

**Figure 3:**
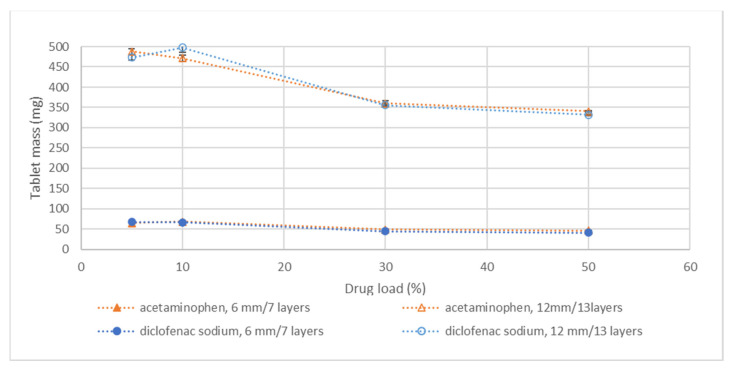
Tablet mass versus drug load for both acetaminophen and diclofenac sodium formulations. The tablets were printed according to the formulations and print settings as provided in Table 4, meaning that the 50% *w*/*w* acetaminophen formulation contains 0.5% *w*/*w* silica and the 30 and 50% *w*/*w* diclofenac sodium formulation contains coarse API and were printed without outer line. Please note that standard deviations for all data points are presented but not visible for the 6 mm/7-layer tablets. In this case, all values of standard deviation were <2.5 mg. The complete dataset of all drug loads can be found in the supplementary information Figure S1.

Tablet mass is directly related to tablet dose and is, therefore, an important parameter to study in case of variations in drug load or tablet dimensions. Increasing tablet dimensions by increasing tablet diameter or by printing additional layers resulted in all cases in an increased tablet mass (Figure 3).

A reduction in tablet mass was observed with increasing drug load for all the printed dimensions (Figure 3). In powder bed printing, the blend is automatically dispersed from a hopper onto the powder platform and smoothed with a counter-rotating roller instead of being compressed. Therefore, bulk density is often related to tablet mass, as in this case. For example, when increasing the acetaminophen load from 5 to 50%, the blend bulk density decreased from 0.61 to 0.35 g/mL and tablet mass reduced from 148.5 to 93.2 mg (9 mm/7-layer tablet). For diclofenac sodium, a decrease in blend bulk density was also observed with increasing drug load from 5 up to 50 % *w*/*w* (0.68 to 0.52 g/mL). Nevertheless, blend bulk density was not the only factor influencing tablet mass. As explained previously, bleeding and densification after wetting are also factors that can affect the mass of the tablet.

For diclofenac sodium, the API grade and print pattern varied in the study setup. The 5 and 10% drug load contained a fine grade of API and the print pattern contained an outer line. The 30 and 50% formulation contained the coarse grade and no outer lines (as indicated in Table 4). This variation resulted also in a variation in tablet mass, being that the coarse grade without a rim had, in general, a lower mass than the fine grade printed with a rim for the 10% drug load formulation (data provided in supplementary information, Figure S1). This is similar to the observations of Section 3.3 regarding variation in print settings.

In conclusion, to avoid density issues, varying the dose of a 3D-printed formulation by varying tablet dimensions is the most straightforward method. However, it is also possible to increase dosing strength by increasing drug load.

### 4.2. Tablet Tensile Strength

Tablet tensile strength is an important indicator of the ability of the dosage form to withstand packaging and remain intact when handled by the patient. Powder bed printed tablets are, in general, more friable than compressed tablets due to increased surface roughness, which results from agglomeration. De-dusting processes and specific packaging can overcome this friability issue.

**Figure 4:**
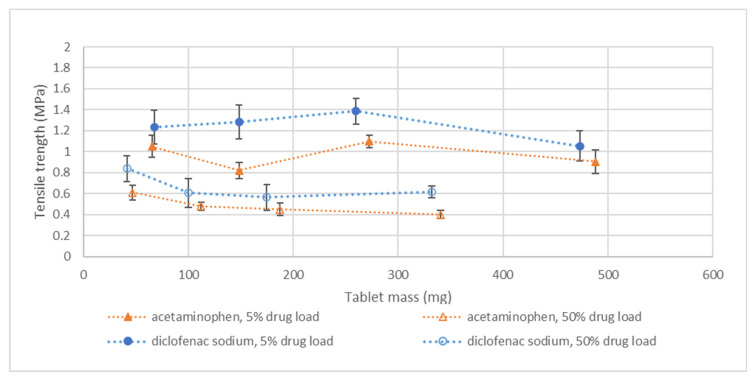
Tensile strength versus tablet mass for both the acetaminophen and diclofenac sodium formulations. Tablet mass was increased by printing the blend at a tablet diameter of 6, 9, and 12 mm. The 12 mm tablet was further increased in mass by increasing the number of layers from 7 to 13. Correlation between print dimensions and tablet mass can be found in Figure 3. The tablets were printed according to the formulations and print settings as provided in Table 4, meaning that the 50% *w*/*w* acetaminophen formulation contains 0.5% *w*/*w* silica and that the 50% *w*/*w* diclofenac sodium formulation contains coarse API and were printed without outer line. The complete dataset of all drug loads can be found in the supplementary information, Figure S2.

Figure 4 shows the tensile strength versus tablet mass profiles. The tablet mass was controlled by increasing the diameter and the number of layers. For a series of tablets with constant drug loading and variable tablet mass, the tensile strength remained fairly constant, with values always being larger than 0.2 MPa (Figure 4). The binding properties of the same formulations were expected to remain equal regardless of tablet size. This effect was observed for both the hydrophilic and hydrophobic model compounds.

When the drug load in the blend was increased, a reduction in tensile strength was observed. Tablet tensile strength remained constant at a 5–10% *w*/*w* API level but decreased at higher loading (Figure 4). This can be explained by the fact that APIs presumably have fewer binding properties than lactose, which acts as a weak binder when wetted. The trends were comparable for both model compounds as the binding properties were not related to their individual solubilities but to their binding ability, which was low for both components.

The API particle size and print settings were varied for the 30–50% *w*/*w* diclofenac sodium blends in order to obtain sufficient flow and wettability (as provided in Table 4). In order to study the effect on tensile strength of fine-particle-size diclofenac sodium with outer line and coarse-particle-size diclofenac sodium without outer line, the 10% diclofenac sodium formulation was printed with both formulations (data in supplementary information, Figure S2). It was observed that the tensile strength of the fine diclofenac sodium was generally higher compared to that of the coarser grade, for example, 1.5 MPa for fine grade versus 1 MPa for coarse grade (6 mm diameter/7-layer print setting). It is possible that the finer API could either directly promote better binding than the coarser API by creating a more compact bed or it interfered less with formation of a binding matrix by the starch.

### 4.3. API Release

In phase 1 and 2a clinical studies, the effectiveness and safety of new drug compounds are studied. Therefore, it is important that the API is completely released at comparable rates across formulations. It is typically required to have more than 80% *w*/*w* drug release in 30 min.

**Figure 5:**
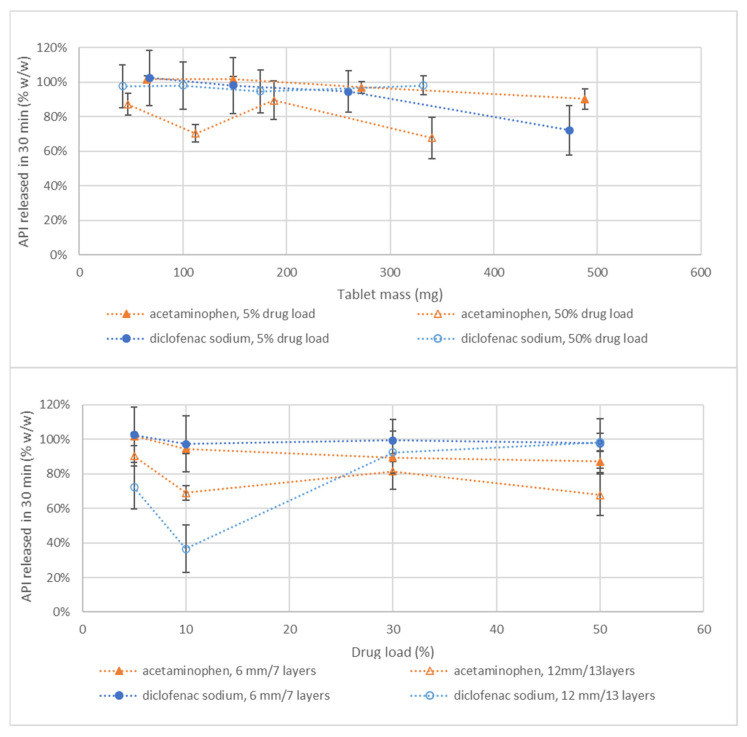
Amount of API released after 30 min versus tablet mass (**upper** panel) and drug load (**lower** panel) for both the acetaminophen and diclofenac sodium formulations. Tablet mass was increased by printing the blend at a tablet diameter of 6, 9, and 12 mm. The 12 mm tablet was further increased in mass by increasing the number of layers from 7 to 13. Correlation between print settings and tablet mass can be found in Figure 3. The tablets were printed according to the formulations and print settings as provided in Table 4, meaning that the 50% *w*/*w* acetaminophen formulation contains 0.5% *w*/*w* silica and the 30 and 50% *w*/*w* diclofenac sodium formulation contains coarse API and were printed without outer line. The complete dataset of all drug loads can be found in the supplementary information, Figure S3.

Figure 5 (upper panel) shows the percentage of API released in 30 min versus the tablet mass. The dissolution data showed that API release was dependent on API type and particle size. For all formulations, a cloud formation of the starch binder was observed as a result of the mild dissolution stress (low impeller speed) applied during the test. This cloud formation resulted in a higher RSD for the released API during dissolution as it is likely that the API was released irregularly from the viscous binder cloud. In general, the dissolution rate of both APIs decreased with increasing tablet mass (Figure 5). This is a result of the larger cloud formation of the starch with larger tablets and the consequently longer diffusion times of the water and API through both tablet and starch clouds. In the case of diclofenac sodium, the effect was more pronounced with the fine-grade API used in the 5% drug load formulation. Although micronized API should provide a quick dissolution, it is also likely to remain more easily entrapped in the binder cloud, thus delaying diffusion and dissolution.

API loading had a limited impact on dissolution for the coarse-grade diclofenac sodium used in the 30 and 50% formulations (Figure 5 lower panel). Usage of fine-grade did result in a decrease in dissolution when the drug load was increased from 5 to 10%. This is probably owing to the cloud formation and sticky behavior of this API, as explained in the previous paragraph. For acetaminophen, the trend is less straightforward as some outliers (e.g., 112 mg, 70% *w*/*w* API release 50% *w*/*w* drug loading) released below 80% *w*/*w* (Figure 5 lower panel). This is probably caused by the sensitive method with a low impeller speed, which results in insufficient movement of the dissolution medium to disrupt the starch matrix.

The lactose/starch platform formulation was suitable for printing formulations ranging from 50 to 500 mg tablet mass, incorporating both hydrophilic and hydrophobic drugs. The tensile strength was constant over the various tablet masses when the drug concentration was constant in the blend. Overall, most tablet formulations met the requirements for rapid dissolution across different API loadings and tablet masses. In the case of diclofenac sodium tablets with the highest tablet mass, longer dissolution times were obtained, but these formulations could be further optimized by varying the print settings in future research.

## 5. Conclusions

Formulations to be used in clinical trials typically have some challenging pre-requisites that need to be met: (i) the possibility to change both API concentration and tablet mass, (ii) physical robustness of the dosage form (to be able to withstand handling), and (iii) the ability to meet immediate release requirements. The work presented in this article demonstrates how powder bed printing can be successfully used to develop flexible platform formulations that are suitable for clinical trials. Tablets with a broad range of drug loadings and tablet masses, with sufficient tablet hardness and yet immediate drug release, could be produced for two different model APIs using conventional tableting excipients.

The clinical trial platform formulation was based on commonly used excipients lactose monohydrate and partially pregelatinized starch. The solubility of API plays an important role in selection of the appropriate partially pregelatinized starch. Different partially pregelatinized starches were not interchangeable because they exhibited different binding/disintegration properties.

The lactose/starch platform formulation was successfully optimized to enable formulation of tablets with drug loading ranging between 5 and 50% *w*/*w* for both model APIs used. The flow and liquid penetration time of the blends are critical material attributes for powder bed printing. For formulation of tablets containing up to 50% *w*/*w* API, the flow and wetting properties of the blends were successfully improved by either selecting a coarser grade of API or by addition of a glidant.

By applying the above considerations, printed tablets with a tensile strength above 0.2 MPa and having an API release of >80% *w*/*w* within 30 min have been created using the platform formulation of lactose/partially pregelatinized starch. For both APIs, printed tablets were achieved at drug levels from 5 up to 50% *w*/*w* and reaching tablet masses from 50 up to 500 mg, thus enabling the dose variation required for clinical trial formulations. Altogether, this research shows that powder-based 3D tablet printing is a realistic option for creating clinical trial tablet DoEs from a technical prospective.

## Figures and Tables

**Table 1:** Overview of print dimensions and amount of print ink per formulation. The print pattern is provided in Figure 1.

LS (mm)	Outer Line	Print Setting (Diameter, Number of Layers)	Amount of Print Ink/Tablet (Gram)
0.35	yes	6 mm, 7 layers	0.039
0.35	no	6 mm, 7 layers	0.030
0.28	yes	9 mm, 7 layers	0.132
0.43	yes	9 mm, 7 layers	0.060
0.35	yes	9 mm, 7 layers	0.083
0.35	no	9 mm, 7 layers	0.075
0.35	yes	12 mm, 7 layers	0.149
0.35	no	12 mm, 7 layers	0.136
0.35	yes	12 mm, 13 layers	0.277
0.35	no	12 mm, 13 layers	0.250

**Table 2:** Impact of different starch grades on tablet hardness and dissolution rate. Top (screening experiment): tablets were printed and analyzed via in-house screening method. Bottom: tablets printed at TNO with a line spacing of 0.35 mm and analyzed as provided in material and methods. nm = not measured. Dissolution rate is indicated as % *w*/*w* API released after 30 min dissolution testing.

		Acetaminophen (×50 = 21 µm)	Diclofenac Sodium (×50 = 7 µm)
Screening test (n = 2)			
20% *w*/*w* starch grade 1	Hardness % dissolution after 30 min	29 N 100%	56 N 91%
20% *w*/*w* starch grade 2	Hardness % dissolution after 30 min	69 N 82%	48 N 45%
Tablets printed at TNO (hardness n = 10, dissolution n = 6)			
20% *w*/*w* starch grade 1	Hardness % dissolution after 30 min	nm nm	52 N 92%
20% *w*/*w* starch grade 2	Hardness % dissolution after 30 min	50 N 93%	51 N 53%

**Table 3:** Tablet tensile strength versus release after 30 min for tablets printed with a diameter of 9 mm and a thickness of 3.1–3.6 mm (corresponding to 7 layers). nm = not measured. Formulations contain 10% API, 20%, starch and 70% lactose.

Description	Amount of Liquid per Tablet (gram)	Acetaminophen ×50 = 21 Micron Tablet Mass (mg)—Tensile Strength (MPa)—% *w*/*w* Released after 30 min	Diclofenac Sodium ×50 = 7 Micron Tablet Mass (mg)—Tensile Strength (MPa)—% *w*/*w* Released after 30 min
LS 0.28—outer line	0.1323	215.0—1.0—78%	nm
LS 0.35—outer line	0.0833	149.5—1.1—93%	147.6—1.2—92 %
LS 0.43—outer line	0.0602	123.1—0.7—100%	nm

**Table 4:** Platform formulation and print settings used for the clinical trial set-up formulations. Lactose monohydrate type A is a sieved grade with an ×50 of 75–85 µm and lactose monohydrate type B is a milled and classified lactose with an ×50 of 80–90 µm. Lactose grades were selected based on previous research [29].

	Hydrophilic Drug Load 5–10–30% *w*/*w*	Hydrophobic Drug Load 5–10% *w*/*w*
**Print setting**	LS 0.35 mm	LS 0.35 mm
**API**	acetaminophen ×50 = 21 micron	diclofenac sodium ×50 = 7 micron
**Starch**	20% *w*/*w* Grade 2	20% *w*/*w* Grade 1
**Filler**	Lactose type A	Lactose type B
**Additive**	None	none
	**Hydrophilic** **Drug Load 50% *w*/*w***	**Hydrophobic** **Drug Load 30–50% *w*/*w***
**Print setting**	LS 0.35 mm	LS 0.35 mm, no outer line
**API**	acetaminophen ×50 = 21 micron	diclofenac sodium ×50 = 24 micron
**Starch**	20% *w*/*w* Grade 2	20% *w*/*w* Grade 1
**Filler**	Lactose type A	Lactose type B
**Additive**	0.5% *w*/*w* silica	none

## Data Availability

Not applicable.

## Supplementary Materials

The following supporting information can be downloaded at: https://www.mdpi.com/article/10.3390/pharmaceutics14112320/s1, Figure S1: Tablet mass versus drug load for both the hydrophilic (upper panel) and hydrophobic (lower panel) model compound formulation. Acetaminophen with an ×50 = 21 micron was used for all formulations. The 10% hydrophobic formulation was printed with both formulations mentioned in Table 4; Figure S2: Tensile strength versus tablet mass for both the hydrophilic (upper panel) and hydrophobic model (lower panel) compound formulations. Tablet mass was increased by printing the blend at a tablet diameter of 6, 9, and 12 mm. The 12 mm tablet was further increased in mass by increasing the number of layers from 7 to 13. Correlation between print dimensions and tablet mass can be found in Figure 3. Acetaminophen with an ×50 = 21 micron was used for all formulations. The 10% hydrophobic formulation was printed with both formulations mentioned in Table 4; Figure S3: Amount of API released after 30 min versus tablet mass for both the hydrophilic (upper panel) and hydrophobic (lower panel) model compound formulations. Tablet mass was increased by printing the blend at a tablet diameter of 6, 9, and 12 mm. The 12 mm tablet was further increased in mass by increasing the number of layers from 7 to 13. Correlation between print settings and tablet mass can be found in Figure 3. Acetaminophen with an x50=21 micron was used for all formulations. The 10% hydrophobic formulation was printed with both formulations mentioned in Table 4; Figure S4: Photograph of printed formulations as provided in Table 4.
