# Supplementary material for: 3D-Powder-Bed-Printed Pharmaceutical Drug Product Tablets for Use in Clinical Studies
Source: Pharmaceutics. 2022 Oct 28;14(11):2320. doi: 10.3390/pharmaceutics14112320 (PMC9699453; doi:10.3390/pharmaceutics14112320)
Supplement: Supplementary file 1 [file pharmaceutics-14-02320-s001.zip › pharmaceutics-1957409-supplementary.pdf]

Supplementary Figures File for 3D-Powder-Bed-Printed Pharmaceutical Drug Product Tablets for Use in Clinical Studies

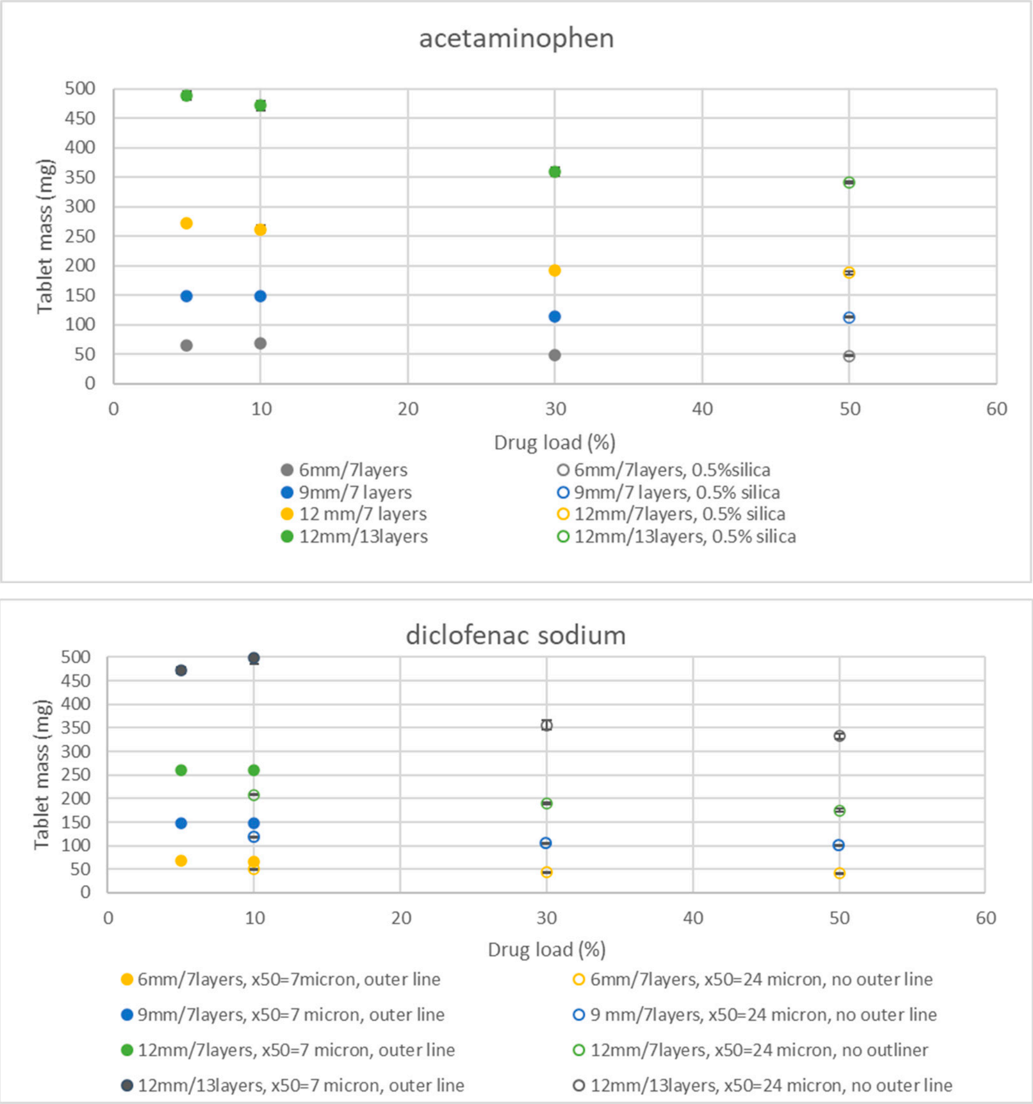

Figure S1. Tablet mass versus drug load for both the hydrophilic (upper panel) and hydrophobic (lower panel) model compound formulation. Acetaminophen with an x50=21 micron was used for all formulations. The 10% hydrophobic formulation was printed with both formulations mentioned in Table 4.

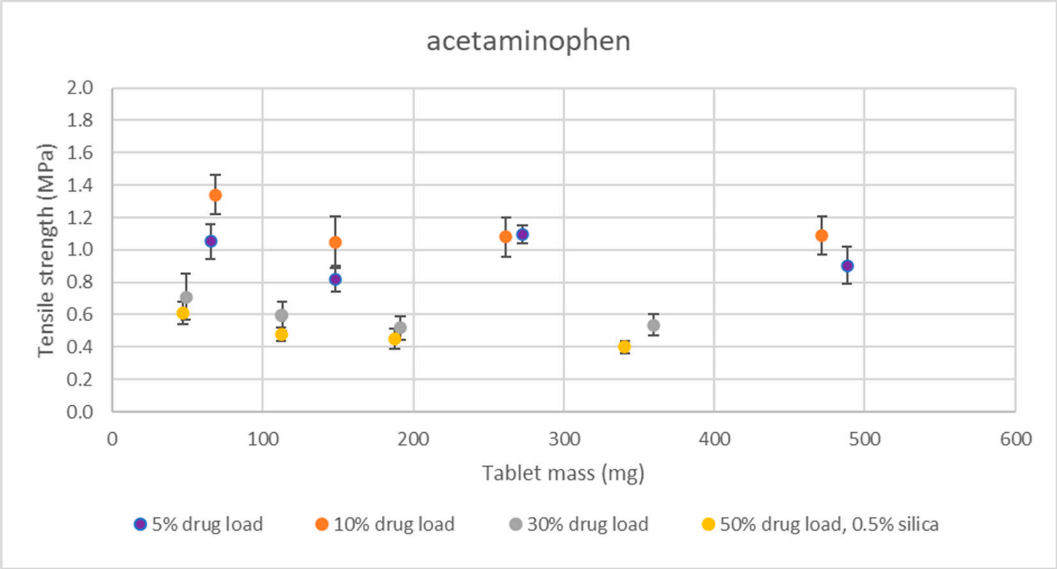

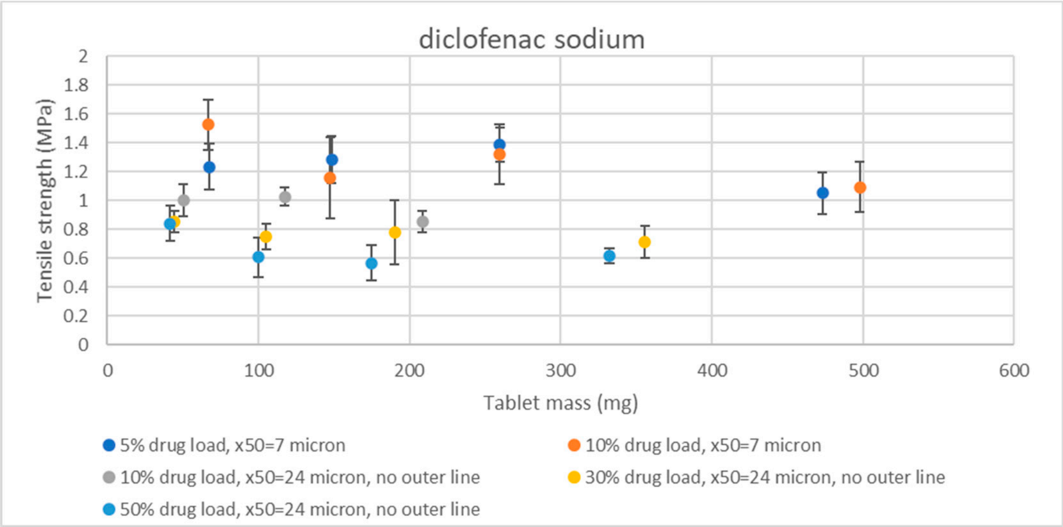

Figure S2. Tensile strength versus tablet mass for both the hydrophilic (upper panel) and hydrophobic model (lower panel) compound formulations. Tablet mass was increased by printing the blend at a tablet diameter of 6, 9, and 12 mm. The 12 mm tablet was further increased in mass by increasing the number of layers from 7 to 13. Correlation between print dimensions and tablet mass can be found in Figure 3. Acetaminophen with an x50=21 micron was used for all formulations. The 10% hydrophobic formulation was printed with both formulations mentioned in Table 4.

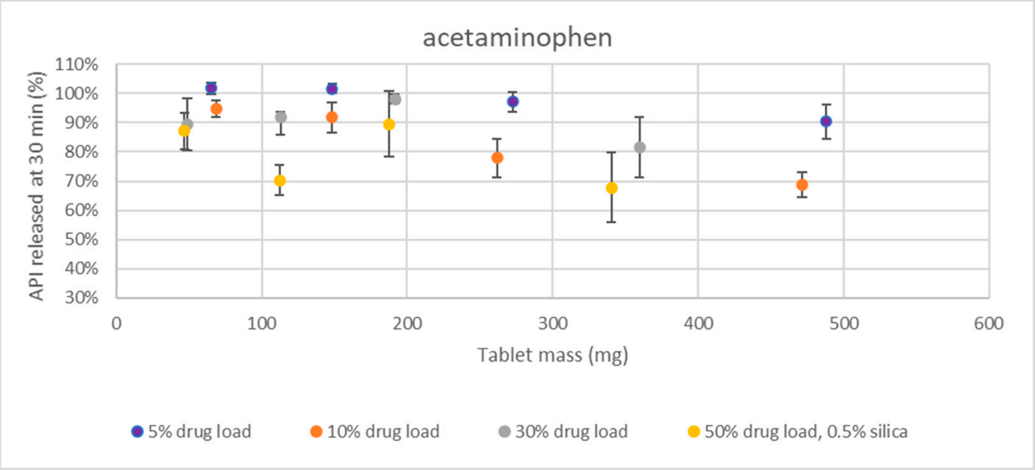

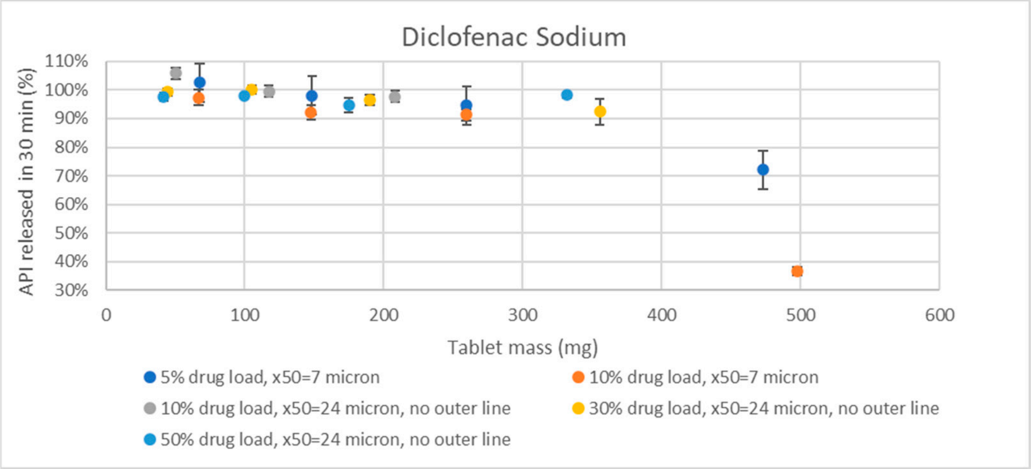

Figure S3. Amount of API released after 30 min versus tablet mass for both the hydrophilic (upper panel) and hydrophobic (lower panel) model compound formulations. Tablet mass was increased by printing the blend at a tablet diameter of 6, 9, and 12 mm. The 12 mm tablet was further increased in mass by increasing the number of layers from 7 to 13. Correlation between print settings and tablet mass can be found in Figure 3. Acetaminophen with an x50=21 micron was used for all formulations. The 10% hydrophobic formulation was printed with both formulations mentioned in Table 4.

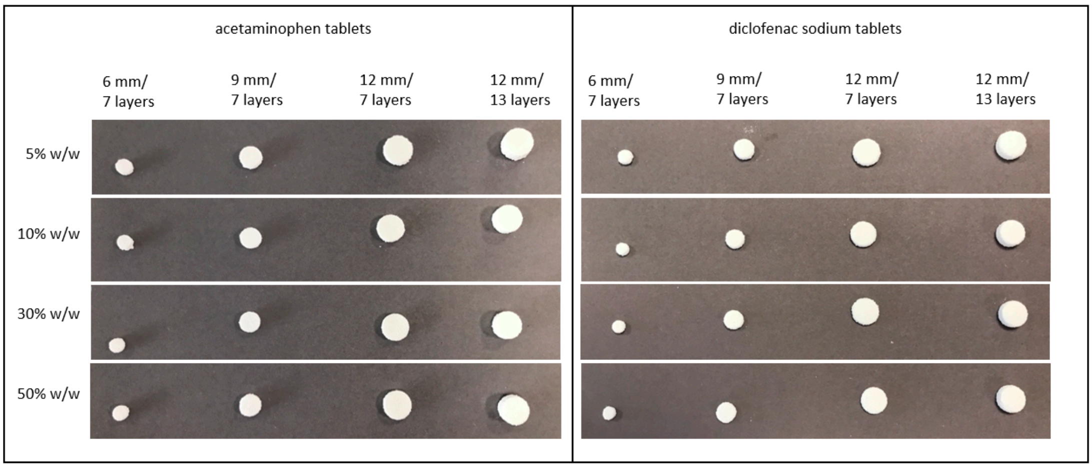

Figure S4. Photograph of printed formulations as provided in Table 4.
